# Potential roles of selected forage grasses in management of fall armyworm (*Spodoptera frugiperda*) through companion cropping

**DOI:** 10.1111/eea.13083

**Published:** 2021-07-22

**Authors:** Duncan Cheruiyot, Xavier Chiriboga Morales, Frank Chidawanyika, Toby J. A. Bruce, Zeyaur R. Khan

**Affiliations:** ^1^ International Centre of Insect Physiology and Ecology (icipe) PO Box Nairobi 30772‐00100 Kenya; ^2^ Department of Zoology and Entomology University of the Free State PO Box 339 Bloemfontein 9300 South Africa; ^3^ School of Life Sciences Keele University Staffordshire ST5 5BG UK

**Keywords:** pest management, fall armyworm, forage grasses, larval performance, oviposition, push–pull, trap plants, Lepidoptera, Noctuidae, crop phenology, companion cropping, *Spodoptera frugiperda*

## Abstract

Production of maize, *Zea mays* L. (Poaceae), in sub‐Saharan Africa is threatened by a new invasive pest, fall armyworm (FAW), *Spodoptera frugiperda* (JE Smith) (Lepidoptera: Noctuidae). To mitigate this threat, push–pull companion cropping, a system originally developed for management of lepidopteran stemborers, may be used to control FAW. The original system involved trap crops that functioned as a ‘pull’ component to attract moths away from the main crop. How grass species can be used as trap crops in a push–pull system to control FAW is a question that remains to be answered, because maize is already a highly preferred host plant. Therefore, we tested oviposition preference of FAW female moths in no‐choice and two‐choice experiments and larval performance on six selected grasses (Poaceae) to assess their roles as trap crop ‘pull’ plants in the system. In no‐choice tests, numbers of eggs deposited on *Brachiaria brizantha* (Hochst. ex A. Rich.) R. Webster cv. ‘Piata’, cv. ‘Mulato II’, and cv. ‘Xaraes’, and Napier grass (*Pennisetum purpureum* K. Schumach) cv. ‘South Africa’ were not statistically different from those deposited on maize. In two‐choice tests between grasses and maize, there were no significant differences in number of eggs laid when the plants were of the same size. However, in two‐choice tests with maize plants half of the size of the grasses, significantly more eggs were laid on *B. brizantha* cv. Xaraes and *P. purpureum* cv. South Africa than on maize, suggesting that crop phenology could make a difference. Numbers of larvae arrested on grass leaf cuts were considerably lower than those on maize leaf cuts after 48 h. In two‐choice tests with maize, molasses grass (*Melinis minutiflora* P. Beauv.) was the only grass that was significantly preferred to maize for larval settlement after 24 h. After 48 h in the two‐choice test, it was the only grass that retained larvae, although the larval count was significantly lower than on maize. Our data show that none of the grasses tested were strongly preferred to maize, but the results indicate plants attractive to FAW adults and larvae that could be utilized in a multiple trap crop approach to target various stages of the pest. Furthermore, results indicate the importance of planting these companion plants earlier than maize.

## Graphical abstract

We tested promising forage grasses (Poaceae) to determine their functions as trap crop ‘pull’ components in a ‘push–pull’ strategy. Although there was no strong preference of the grasses to maize, we observed that crop phenology could make a difference. The study identifies plants attractive to adults and larvae of FAW (Lepidoptera: Noctuidae) that could be utilized in a multiple trap crop approach to target various stages of the pest. The importance of planting these companion plants earlier than maize is shown.

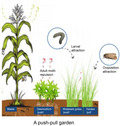

## Introduction

Fall armyworm (FAW), *Spodoptera frugiperda* (JE Smith) (Lepidoptera: Noctuidae), is an economically important pest of maize, *Zea mays* L. (Poaceae), with up to 353 recorded larval host plant species in 76 families (Montezano et al., 2018). In Africa, it was first reported in central and western regions in 2016 (Goergen et al., 2016), and in most of sub‐Saharan Africa by 2017 (Day et al., 2017) where it has become the most important crop pest of maize (Kumela et al., 2019). Maize is a staple food crop in Africa, where over 200 million people depend on it for food and nutritional security. It accounts for almost half of the calories and protein consumed in eastern and southern Africa, and one‐fifth in West Africa (Macauley, 2015). With the demand for maize in the developing world, where population growth is projected to double by the year 2050 (Rosegrant et al., 2009), maize yield losses due to FAW will exacerbate challenges in meeting the growing demand. For example, FAW is estimated to cause 34% loss in maize yield, translating to more than 1 million tons of maize lost in Kenya alone (De Groote et al., 2020). This is a major shock to food supply and it affects the already fragile economic situation of many households across sub‐Saharan Africa.

Forage grasses are recognized as valuable sources of fodder for cattle in smallholder crop‐livestock farming systems in sub‐Saharan Africa (Ates et al., 2018; Paul et al., 2020). Some grass species in the Poacea family – such as *Brachiaria brizantha* (Hochst. ex A. Rich.) R. Webster cv. ‘Mulato II’, Napier grass (*Pennisetum purpureum* K. Schumach), and molasses grass (*Melinis minutiflora* P. Beauv.) – possess useful properties important in management of cereal pests through push–pull companion cropping systems (Khan et al., 2010). Push–pull is a conservation agriculture technology developed by the International Center of Insect Physiology and Ecology (*icipe*, Kenya) in collaboration the Rothamsted Research (UK) and national partners for integrated management of insect pests, weeds, and soil health in Africa (Cook et al., 2007; Khan et al., 2010, 2011). In the original version of push–pull, maize is intercropped with desmodium (*Desmodium uncinatum* Jacq.) (push) as an adult stemborer repellent plant, and surrounded by Napier grass (pull) as an adult stemborer attractant border plant (Cook et al., 2007; Khan et al., 2010). Drought‐tolerant companion plants, such as *B. brizantha* cv. Mulato II, have been identified and incorporated in various versions of the push–pull technology with improved efficiency and sustainability in diverse agro‐ecological conditions (Midega et al., 2015; D Cheruiyot, JO Pitchar, CAO Midega, J Van den Berg, JA Pickett & ZR Khan, unpubl.).

The roles of forage grasses as companion plants in push–pull have been documented, particularly for the lepidopterous stemborers *Chilo partellus* (C. Swinhoe) and *Busseola fusca* (Fuller). Napier grasses and Sudan grass, *Sorghum sudanensis* (Pers.), are highly preferred hosts for oviposition by female stemborer moths. Additionally, Napier grass is disadvantageous to survival of their larvae (Khan et al., 1997, 2000; Van Den Berg, 2006); hence, both grass species are suitable trap plants for the pest. In response to penetration by early larval stages, Napier grass produces a sticky substance that impedes larval development (Khan et al., 2000). In contrast, Molasses grass is not suitable for both oviposition and larval development (Khan et al., 2000), and was used as a repellent intercrop. Some *Brachiaria* species have been observed to support oviposition by *C. partellus* (Chidawanyika et al., 2014; Cheruiyot et al., 2018), despite being detrimental to larval survival (Midega et al., 2011; Cheruiyot et al., 2018). Furthermore, *B. brizantha* functions as a signal grass in a push–pull system. When *C. partellus* moths deposit eggs on their leaves, *B. brizantha* becomes more attractive to an indigenous and highly adapted natural enemy of the herbivore, the larval parasitic wasp *Cotesia sesamiae* Cameron (Bruce et al., 2010).

Push–pull technology has also been observed to be effective against FAW (Khan et al., 2018; Midega et al., 2018). Current evidence suggests that desmodium intercrop remains repulsive, but a peripheral *B. brizantha* cv. Mulato II may not be effective trap plants for FAW (A Tamiru, IS Sobhy, XC Morales, D Nyagol, S Subramanian, CAO Midega, TJA Bruce & ZR Khan, unpubl.). Here, we assessed FAW adult oviposition preference and subsequent larval performance on six forage grasses in order to gauge their potential for use as trap plants for FAW. Plant selection was based on reports in previous studies including agronomic performance (biomass yield, tolerance to drought, and resistance to the red spider mite *Oligonychus trichardti* Meyer), potential for use as attractive plants in pest management strategies, and farmers’ opinions (Khan et al., 2000, 2006; Van Den Berg, 2006; Midega et al., 2011; Cheruiyot et al., 2018; Cheruiyot et al., 2020).

## Materials and methods

### Study site

The study was carried out at the International Center of Insect Physiology and Ecology, Thomas Odhiambo Campus (*icipe*‐TOC), Mbita Point (0°25′S, 34°12′E, 1 200 m a.s.l.) under both laboratory (24 ± 1 °C, 70 ± 5% r.h.) and semi‐field screenhouse (28 ± 3 °C, 65 ± 5% r.h.) conditions. The field station is located on the shores of Lake Victoria in western Kenya.

### Test plants and insects

Six forage grasses – comprising two Napier grass (*P. purpureum*) varieties, cvs. ‘Ouma II’ and ‘South Africa’; three brachiaria grass (*B. brizantha*) varieties, cvs. ‘Xaraes’, ‘Piata’, and ‘Mulato II’; and molasses grass, *M. minutiflora* – were selected from a larger collection of grasses maintained at *icipe*‐TOC. As maize is a preferred host for FAW, a maize hybrid (H507) was used as the control for experiments. All the grass plants were grown from root splits in 5‐l plastic pots with vertisol soil from the fields at Mbita Point, whereas maize plants were grown from seeds in similar pots filled with the same soil. No fertilizer was added. Fall armyworm reared for one generation from larvae collected from maize in the field at *icipe‐*TOC insectary using a previously described artificial diet for FAW (Prasanna et al., 2018) and female moths were used in experiments.

### Oviposition bioassays

Oviposition tests were conducted following modifications of procedures described by Khan et al. (2007) and Midega et al. (2011). In no‐choice tests, 3‐ to 4‐week‐old seedlings of each test plant were placed in oviposition cages (80 × 40 × 40 cm) covered with fine wire mesh netting and replicated 10×. A 10‐cm‐diameter wad of cotton wool moistened with water was placed in each cage for the moths to feed on. Five gravid naive moths were introduced into each cage before dusk and allowed to oviposit for 48 h under natural light conditions of L12:D12.

For two‐choice tests, potted plants of each of the grass species and maize were placed in opposite corners of each cage with 10 replications. Three categories of two‐choice tests were conducted, based on the size of the plants: (1) grass vs. maize plant twice the size of the grass (V4 stage of maize), (2) grass vs. maize plant of the same size of the grass (V3 stage of maize), and (3) grass vs. maize plants half of the size of the grass (V2 stage of maize). Vegetative (V) stage of the maize is defined as the number of maize leaves displaying a leaf collar and not the total number of visible leaves on the plant; i.e., a V3 maize seedling has three leaves displaying leaf collars (Prasanna et al., 2018). Grass species used were 3–4 weeks old. A 10‐cm‐diameter wad of cotton wool moistened with water was placed in each cage for the moths to drink from. Five gravid naive moths were introduced into the cage before dusk and allowed to oviposit for 48 h under natural light conditions of L12:D12.

Plants were assessed for FAW eggs after the oviposition period. Fall armyworm eggs are sometimes deposited in layers or with layers of grayish scales between the eggs and over the egg mass with a furry or moldy appearance, making it difficult to count. The number of eggs was therefore estimated using a formula we developed by regressing the weight and number of eggs in a sample of 20 single‐layered egg masses. The regression coefficient (r^2^ = 0.91) was highly significant (P<0.001), and the regression equation was y = 13.5x–6.9, where x is the weight (mg) of egg mass and y the calculated number of eggs in the egg mass.

### Larval preference bioassays

#### Arrestment and dispersal of first instars

This experiment was conducted following the procedures described by Khan et al. (1997). A 6‐cm‐long leaf cut from each test plant was placed individually, with the adaxial side facing upwards, in the center of a 9‐cm‐diameter Petri dish lined with moist filter paper. A moist cotton wad was placed at either end of the leaf cutting. Ten first instar FAW were then introduced on top of each leaf cutting. The Petri dishes were covered and sealed with parafilm to prevent larvae from escaping and kept in a dark room. The larvae remaining on the leaf tissue were counted after 1, 24, and 48 h of release. Ten replicates per grass variety were tested in each experiment.

#### Orientation and settling of larvae

A two‐choice test was conducted to determine larval preference of FAW neonate larvae between a maize leafcut and a leafcut of each grass variety following modification of a procedure described Khan et al. (2007). Four 3‐cm‐long leaf cuts of the plant being compared were laid alternately and radially in a 15‐cm‐diameter Petri dish lined with a moist filter paper disc, two of each grass and two of maize, with their adaxial surfaces facing upwards. Ten neonate FAW were introduced at the center of each Petri dish. Petri dishes were covered and sealed with parafilm and kept in a dark room. Larvae were allowed to orientate and settle on their preferred leaf cuts. The larvae on or underneath each leaf cut were counted after 1, 24, and 48 h to determine their orientation and settling preference. Ten replicates per grass‐maize choice combination were performed in an experiment.

#### Leaf area damaged by the larvae

Cut pieces (3 cm long) of the second‐youngest leaf of 3‐week‐old plants were placed in a 6‐cm‐diameter Petri dish lined with wet filter paper to limit desiccation. Each piece of leaf was placed in a different Petri dish. Ten replicates of each grass species were tested and maize leaf cuts were used as a control. Ten neonate larvae were placed on each leaf cut. The Petri dishes were covered and sealed with parafilm to prevent larvae from escaping and kept in a dark room. The leaf area (mm^2^) damaged by the larvae was measured after 48 h using the Bio‐Leaf mobile application (Machado et al., 2016). The application can measure the damaged leaf area and the extent of damage by insects as compared to the normal leaf area. The surface area damaged after feeding indicates feeding levels of the larvae on the leaf tissue.

#### Food assimilation by larvae

A 4‐cm‐long stem segment of each plant species was obtained, weighed (S1), and placed in a glass vial (4.1 × 1 cm) with a piece of moistened cotton wool placed in the base to reduce plant desiccation and to provide water for FAW larvae. Third instars, previously starved for 2 h but water satiated to eliminate effects of desiccation, were also weighed (W1) on a PM460 microbalance (Mettler‐Toledo, Greifensee, Switzerland) before individually introducing them into the vials. The vials were then covered with cotton wool plugs and kept in a dark room for 24 h after which the larvae and excreta were removed from the stem and the non‐consumed parts of the stem segments were weighed again (S2). To determine weight loss due to evaporation, 10 stem segments (4 cm long) of each treatment were weighed (CE1), kept in similar vials alongside the experimental ones, and weighed again after 24 h (CE2). The amount of food ingested was determined by computing the difference between the initial and the final weight (S1 – S2) of the stem tissue after adjustment for weight loss due to evaporation (Khan & Saxena, 1985). Each treatment was replicated 10×.

To determine the amount of food assimilated, each larva was weighed again (W2). To determine larval weight loss due to metabolism, 10 larvae were weighed (C1), kept alongside the experiment in similar vials without stem pieces and weighed again after 24 h (C2). The amount of food metabolized by each larva was determined using the equation from Khan & Saxena (1985). The following equation was used to calculate food assimilation:

$$\text{Food assimilated}=W1\times \left(C1‐C2\right)/C1+W2‐W1,$$

where W1 is the initial weight of treated larva, W2 its final weight, C1 the initial weight of control larva, and C2 its final weight.

### Data analysis

Data on two‐choice larval orientation and settlement, and adult oviposition between the grasses and maize were analyzed using an unpaired two‐sample Student’s t‐test. Data on arrest and dispersal, leaf feeding, food ingestion and assimilation, and no‐choice oviposition tests were subjected to one‐way ANOVA using the generalized linear model to test for any differences between treatments. All data were log(x + 1)‐transformed to normalize them and conform to assumptions of tests. Means were separated by Fisher’s least significant difference (LSD) test (α = 0.05). Means of non‐transformed data are presented in figures and tables. Analyses were computed using R v.4.0.2 (R Core Team, 2020).

## Results

### Oviposition bioassays

**Figure 1:**
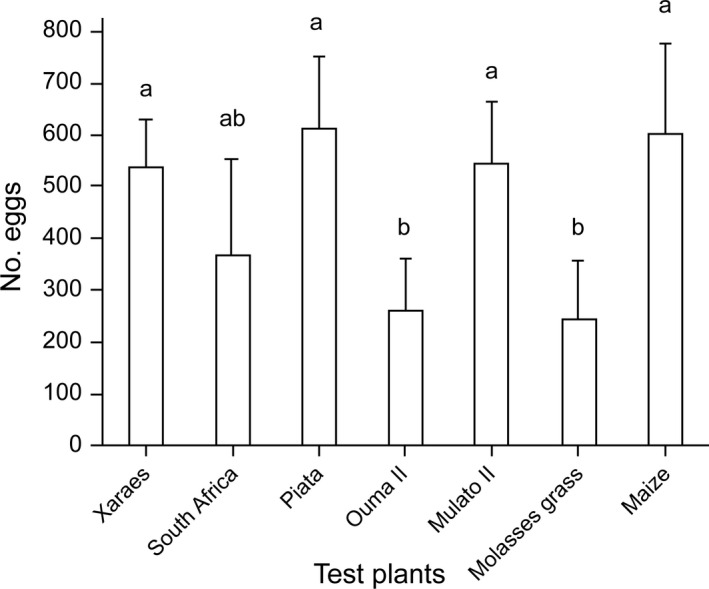
Mean (± SEM; n = 7) number of fall armyworm eggs on various forage grass varieties and maize plants in no‐choice oviposition tests. Means capped with different letters are significantly different (Fisher’s LSD test: P<0.05).

In no‐choice oviposition experiments, differences were found in the number of eggs deposited on the various plants tested (F_6,63_ = 1.33, P = 0.029). The number of eggs deposited on brachiaria grasses (cvs. Piata, Mulato II, and Xaraes) was comparable with that deposited on maize. Meanwhile molasses grass and Napier grass cv. Ouma II were the least oviposited on (Figure 1). Egg numbers deposited on Napier grass cv. South Africa did not differ significantly from those on the other test plants.

**Figure 2:**
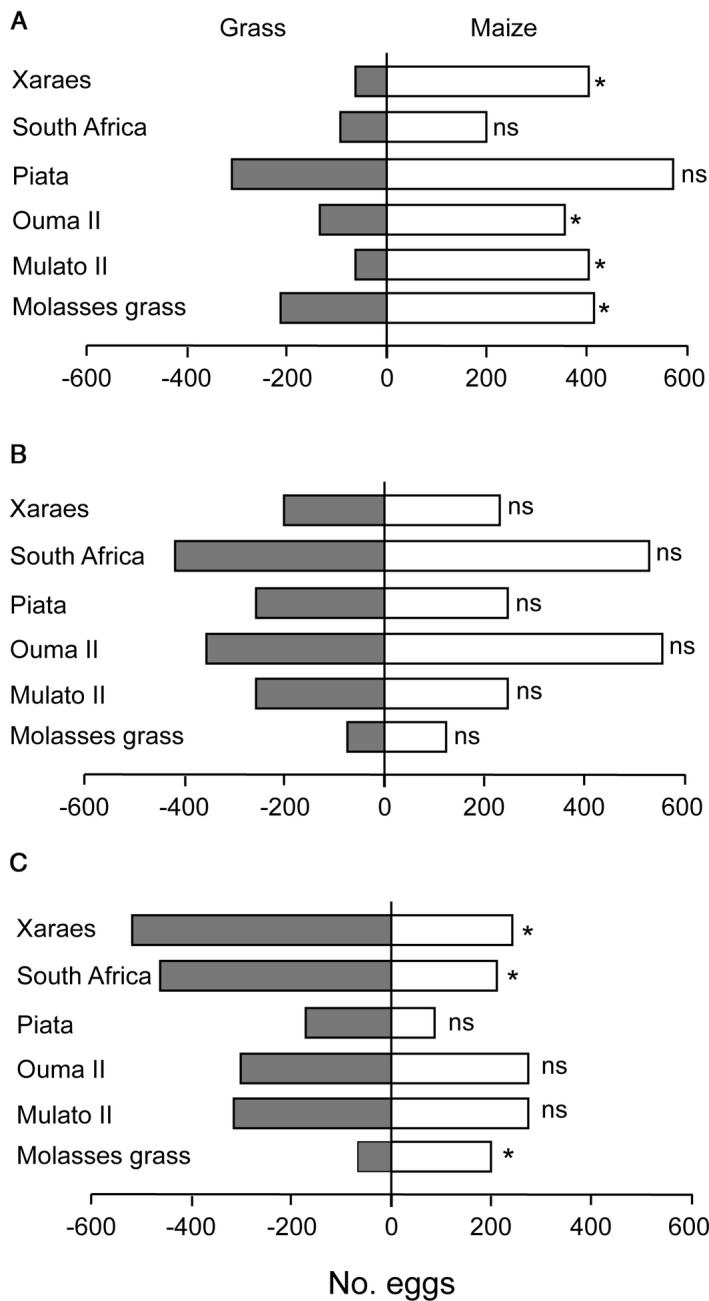
Number of fall armyworm eggs deposited in two‐choice tests between a grass variety and a maize plant (A) twice the size of the grass, (B) of the same size, and (C) half the size of the grass. Asterisks indicate significant preference (t‐test: P<0.05; ns, P>0.05).

In choice tests, results depended on the relative size of plants. When FAW female moths was given a choice between a grass and a maize plant twice the size of the grass, they deposited significantly more eggs on maize plants, except when moths were exposed to *Brachiaria* cv. Piata vs. maize where the numbers of eggs were not statistically different (Figure 2A). When moths were exposed to plants of the same size, numbers of eggs deposited were not statistically different in all combinations (Figure 2B). When grasses were exposed to maize plants half of their sizes, significantly more eggs were laid on *B. brizantha* cv. Xaraes and *P. purpureum* cv. South Africa than on maize plants (Figure 2C).

### Larval arrestment and preference bioassays

Under no‐choice conditions, there were differences among the test plants for the number of first instar FAW retained on leaf cuts after 1 h (F_6,63_ = 24.9), 24 h (F_6,63_ = 21.16), and 48 h (F_6,63_ = 15.4, all P<0.001). The highest numbers of larvae were recorded on leaf cuts of *Brachiaria* cv. Mulato II (9.9 and 6.1), maize (9.2 and 7.4), and molasses grass (8.9 and 8.0) at 1 and 24 h after release, respectively, whereas *Brachiaria* cv. Xaraes had the lowest numbers (2.5 and 1.5, respectively) during the same period (Table 1). At 48 h after release, the most larvae were retained on maize leaf cuts (8.6) followed by molasses grass (6.4), whereas Napier grass cv. Ouma II had the least (0.8).

**Table 1:** Mean (± SEM) number of fall armyworm first instars retained after 1–48 h on leaf cuts of various grasses under no‐choice conditions

Test plant	1 h	24 h	48 h
Mulato II	9.9 ± 0.10a	6.1 ± 0.48a	2.7 ± 0.50c
Maize	9.2 ± 0.25ab	7.4 ± 0.50a	8.6 ± 0.37a
Molasses grass	8.9 ± 0.31ab	8.0 ± 0.21a	6.4 ± 0.50b
Piata	7.7 ± 0.47bc	4.0 ± 0.47b	1.0 ± 0.39d
South Africa	7.3 ± 1.82c	3.7 ± 1.83b	3.6 ± 0.58c
Ouma II	7.2 ± 1.93c	6.6 ± 2.01a	0.8 ± 0.25d
Xaraes	3.5 ± 1.18d	1.5 ± 1.80c	1.4 ± 0.31d

Means within a column followed by the same letter are not significantly different (Fisher’s LSD test: P>0.05).

**Figure 3:**
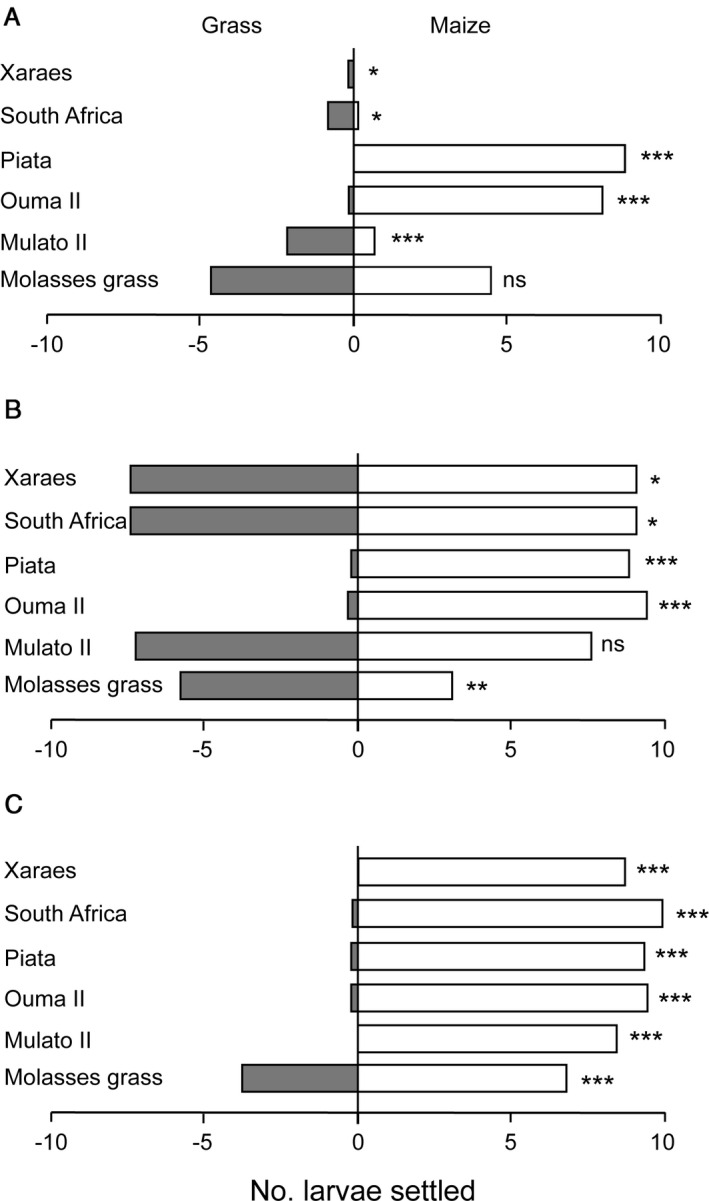
Number of fall armyworm first instars present (A) 1 h, (B) 24 h, and (C) 48 h after infestation in two‐choice tests between a grass variety and a maize plant. Asterisks indicate significant preference (t‐test: *0.01<P<0.05, **0.001<P<0.01, ***P<0.001; ns, P>0.05).

In two‐choice larval orientation and settling tests, after 1 h, leaf cuts of *Brachiaria* cv. Xaraes, Napier grass cv. South Africa, and *Brachiaria* cv. Mulato II had significantly more larvae compared to maize leaf cuts. During the same period, significantly more larvae were recorded on maize than on *Brachiaria* cv. Piata and Napier grasses cv. Ouma II (Figure 3A). After 24 h, a significantly higher number of larvae was recorded on maize leaf cuts compared to *Brachiaria* cvs. Xaraes and Piata and Napier grass cvs. South Africa and Ouma II (Figure 3B). However, a significantly higher number of larvae was recorded on molasses grass compared to maize at the same time. After 48 h, a very strong preference of maize was observed. In all combinations, maize leaf cuts recorded a significantly higher number of larvae compared to the alternative grass species (Figure 3C). The preference for maize was less strong when larvae were offered a choice with molasses grass but nevertheless maize was still preferred to molasses grass after 48 h.

The leaf area damaged by first instars varied among the test plants (F_6,63_ = 13.6, P<0.001) (Table 2). The most damage was observed on maize leaf cuts (10.30 cm^2^), followed by molasses grass (5.48 cm^2^), whereas *Brachiaria* cv. Piata, and *Brachiaria* cv. Xaraes were not damaged at all. There were also differences among the test plants for food ingested (F_6,63_ = 5.62) and food assimilated (F_6,63_ = 58, both P<0.001) by third instar FAW. Interestingly, the amount of stem mass ingested was comparably high for maize (41.58 mg), molasses grass (38.14 mg), *Brachiaria* cv. Mulato II (37.20 mg), *Brachiaria* cv. Xaraes (34.67 mg), and Napier grass cv. South Africa (30.45 mg), and low for *Brachiaria* cv. Piata (9.77 mg) and Napier grass cv. Ouma II (14.85 mg). Significantly more food was assimilated in maize followed by molasses grass and much less for the rest of the test plants (Table 2).

**Table 2:** Mean (± SEM) area of leaf damaged (cm^2^), stem feeding (mg ingested), and food assimilation (mg) by fall armyworm first and third instars on various grasses under no‐choice conditions

Test plant	Leaf area (cm^2^) damaged by five first instars	Stem weight (mg) ingested by a third instar	Food assimilated by a third instar (mg)
Mulato II	0.50 ± 0.12c	37.20 ± 8.06a	3.41 ± 0.72c
Maize	10.30 ± 0.93a	41.58 ± 7.23a	31.28 ± 7.85a
Molasses grass	5.48 ± 0.50b	38.14 ± 4.49a	9.01 ± 1.86b
Piata	0.00 ± 0.00d	9.77 ± 2.78b	4.28 ± 2.44c
South Africa	0.04 ± 0.01d	30.45 ± 5.77a	4.42 ± 0.89c
Ouma II	0.04 ± 0.02d	14.85 ± 2.74b	5.32 ± 0.64bc
Xaraes	0.00 ± 0.00d	34.67 ± 1.67a	2.15 ± 0.76c

Means within a column followed by the same letter are not significantly different (Fisher’s LSD test: P>0.05).

## Discussion

Habitat manipulation is an important agro‐ecological approach for integrated pest management (Gurr et al., 2017). Trap cropping is a habitat management strategy traditionally used in insect pest management, whereby vegetative diversification is used to lure insect pests away from the main crops during a critical period by providing them an alternative preferred host choice (Shelton & Badenes‐Perez, 2006). No‐choice oviposition in our study differed among the tested plants, indicating intra‐ and interspecific variability in FAW host plant ovipositional preference in grasses. Oviposition on the grasses was comparable to that on maize in no‐choice tests. This implies that these grasses may attract gravid moths for oviposition as much as maize. These grasses can be considered as potential ‘pull’ plants for FAW in push–pull technology in the sense that the ‘push’ functionality of push–pull reduces the moths’ preference for maize, giving an advantage to the grasses as preferred hosts for oviposition in the system. However, none of the grasses tested were strongly preferred over maize.

Evidence from our choice test oviposition assays shows there is only a preference for the candidate trap plants if they are considerably larger than the maize. When the grass varieties were compared with maize plants twice their size, more eggs were deposited on maize plants, though the difference was not significant for comparisons involving cv. South Africa and *B. brizantha* cv. Piata. Eggs deposited on test plants of the same size were comparable in all instances. Significantly more eggs were deposited on grass varieties cv. South Africa and *Brachiaria* cv. Xaraes when compared with eggs deposited on maize half their sizes, suggesting that these grass varieties may function as pull plants when they are at least twice as large as maize. This may also suggest that visual cues may be playing a role in host preference for oviposition of which host size is a key factor. Another possibility is that a greater quantity of volatiles is produced by larger grasses, therefore being more attractive than maize. Another possible explanation is that larger plants are of higher quality or simply more apparent in the environment (Knolhoff & Heckel, 2014). The opposite occurred with molasses grass, even though the grass had greater size and biomass compared to maize plants; FAW still preferred to lay eggs on maize. This suggests that molasses may be producing volatiles that are repellent to FAW female moths, and thus may be a candidate repellent intercrop species that could serve as a ‘push’ component in a push–pull system. Preference of larger plants for oviposition by FAW demonstrates the importance of plant phenology in host selection by gravid moths. Our observations support previous findings that found larger plant size in wheat was more attractive for oviposition by the wheat stem sawfly, *Cephus cinctus* Norton (Buteler et al., 2009). Efficiency of companion cropping can thus be enhanced by employing tactics such as planting the companion plants early to the main crop and ensuring the perennial companion plants are fully grown by the time the main crop is planted in subsequent seasons.

Once the pest is attracted to the trap plant, the next requirement for effective trap cropping is retention of the pest on the trap plant, or strategies to prevent the pest from dispersing back to the crop being protected (Holden et al., 2012). Our results indicate that the number of larvae that remained on leaf cuts of all test plants rapidly declined over a period of 48 h, except on molasses grass and maize. This trend is similar with observations of stemborer larvae on some *Brachiaria* spp. (Cheruiyot et al., 2018) and Napier grass varieties (Khan et al., 2006; Van Den Berg, 2006). Results of two‐choice larval orientation tests between the grasses indicate that more larvae settled of leaf cuts of maize than on all the grasses except molasses grass at 24 h after release. Although more larvae settled on maize than on molasses grass after 48 h, molasses grass is the only grass species that retained significantly more FAW larvae compared to other grasses, and the same trend was observed for leaf feeding. Nonetheless, it is evident that molasses grass is the most attractive among the grasses to larvae, and thus it may be considered as an attractive trap plant for FAW larvae in their first instars. Third instars fed on all plants tested, although food assimilation was higher on maize plants followed by molasses grass, and it was very low on the rest of the grasses. The less assimilated grasses are less digestible, probably due to poor nutritive value, or they may have antibiotic effects on FAW larvae (Khan et al., 2006); therefore, survival of FAW larvae on these plants is very unlikely to occur. However, as they do not retain FAW larvae, they cannot function as trap plants for FAW larvae.

In summary, this study demonstrates FAW has a strong preference for maize and this means it is hard to find trap plants that are more attractive to FAW than the maize itself. This shows that it is challenging to develop a ‘pull’ component drawing FAW away from the main maize crop and that success with push–pull companion cropping against the pest most likely relies more on ‘push’ effects with companion crops repelling the pest. Whereas we show only limited suitability of the forage grass species, tested here for their usefulness as trap crops for control of the FAW in a push–pull companion cropping system, we found that the phenology of the trap crop can make a difference. We propose that *B. brizantha* cv. Xaraes and *P. purpureum* cv. South Africa could be used as attractive plants for FAW oviposition if they are planted earlier than the maize crop and are fully grown by the time maize is planted in subsequent seasons. There is therefore a need to conduct trials under field conditions to confirm these observations.

In the case that FAW female moths lay eggs on these grass varieties, the larvae may immediately disperse after eggs hatch. Therefore, we propose the use of another companion plant that arrests FAW larvae, in this case molasses grass. In this model, we propose a row of FAW larval attractive molasses grass should be cropped in between rows of adult attractive plants. This provides an opportunity to exploit multiple trap cropping to enhance the push–pull cropping system to control FAW. Multiple trap cropping involves planting several plant species simultaneously as trap crops with the purpose of either managing several insect pests at the same time or enhancing the control of one insect pest by combining different plants to enhance attractiveness during different stages. There is therefore a need to study the patterns and magnitude of FAW larval dispersal within this mixture of trap plants and between the trap plants and maize. This will guide in designing an appropriate layout for integrating multiple trap crops for enhanced efficiency of push–pull technology. Molasses grass is further known to release volatiles that attract stemborer natural enemies and we hypothesize that it has the same effects with natural enemies of FAW. There is a need for further studies to investigate the tritrophic interactions of the candidate companion plants with FAW and its natural enemies. Our study adds to our knowledge of how graminaceous companion plants may function in a push–pull system against FAW. If there is only a limited trap crop ‘pull’ component, this suggests that current success with push–pull against FAW with a *Brachiaria* cv. Mulato II border crop (Khan et al., 2018; Midega et al., 2018) depends mostly on the ‘push’ activity of repellent intercrops crops, such as desmodium legumes, and the attraction of natural enemies.

## Data Availability

The data that support the findings of this study are available from the corresponding author upon reasonable request.
